# Mechanical Thrombectomy of a Submassive Pulmonary Embolism in the Second Trimester of Pregnancy

**DOI:** 10.7759/cureus.41578

**Published:** 2023-07-08

**Authors:** Dhaval Trivedi, Ruth Minkin

**Affiliations:** 1 Department of Internal Medicine, NewYork-Presbyterian Brooklyn Methodist Hospital, New York City, USA; 2 Department of Pulmonary and Critical Care Medicine, NewYork-Presbyterian Brooklyn Methodist Hospital, New York City, USA

**Keywords:** mfm, pregnancy, right heart strain, mechanical thrombectomy, acute pulmonary embolism

## Abstract

Venous thromboembolism (VTE) represents a potentially severe and infrequent complication that can occur in the pregnant population. The nuance in recognizing and diagnosing this condition can be quite difficult due to the changes that occur during pregnancy. This case highlights the importance of diagnosing pulmonary embolism in pregnancy, classifying the degree of disease, and determining the best treatment for both mother and fetus. Although rare, early diagnosis and treatment are crucial in order to reduce morbidity and mortality.

## Introduction

Venous thromboembolism (VTE) is a rare but serious complication that can exist in the pregnant population. VTE is diagnosed in approximately one in 2,000 pregnancies, with retrospective studies showing incident rates of 0.71 per 1,000 deliveries of deep venous thrombosis (DVT) and 0.15 per 1,000 deliveries of pulmonary embolism (PE) [1]. PE is a leading cause of maternal mortality in pregnancy, accounting for nearly 15% of all maternal deaths [2]. The infrequency of this disease and the serious complications that exist between the fetus and mother in the setting of VTE make the management of VTE difficult and nuanced. Early diagnosis and effective treatment can reduce mortality and improve outcomes for both the fetus and the mother.

## Case presentation

A 33-year-old female, G3P1011 at 24 weeks and three days, with sickle cell trait and uterine fibroids presented to labor and delivery triage for evaluation of progressively worsening dyspnea on exertion for 10 days. Her vitals were notable for tachycardia of 119 beats/minute, a temperature of 36.9°C, a blood pressure of 128/71, a respiratory rate of 20 breaths/minute, and desaturation on room air of 85%, requiring two liters of oxygen via a nasal cannula to maintain saturation >92%. An electrocardiogram (EKG) showed sinus tachycardia. Blood work revealed elevated white blood cells (WBCs) (10.8 x 10^3/uL) and troponin (189 ng/L). A bedside echocardiogram showed right ventricular (RV) dilation with hypokinesis of the right ventricular free wall consistent with right ventricular strain, as evidenced in Video 1.

**Video 1:** The right ventricle is severely dilated with severely reduced systolic function.

**Figure 1:**
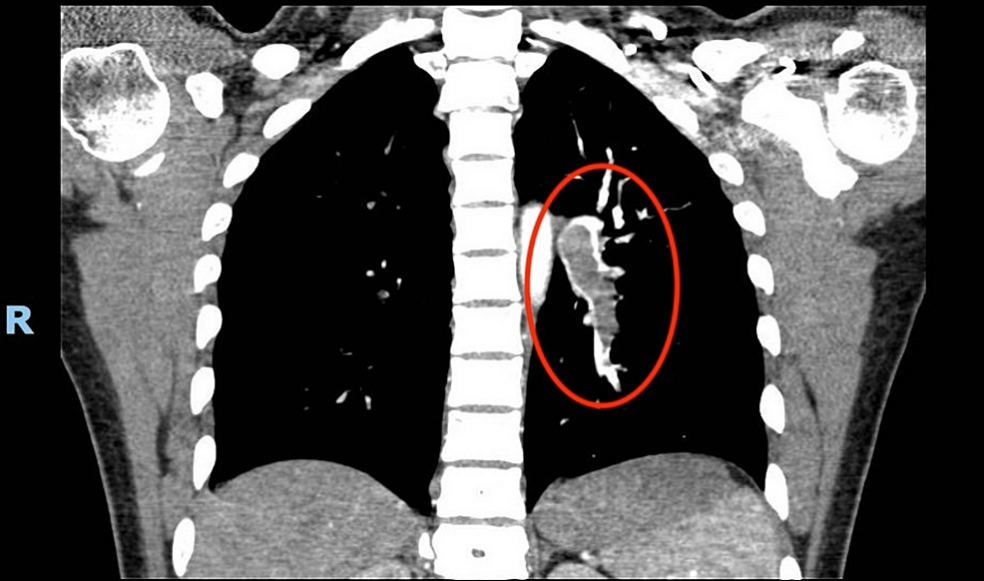
A CT angiogram of the chest (coronal) showing extensive involvement (noted within the red circle) of the left pulmonary artery and subsequent segmental and interlobar arteries.

**Figure 2:**
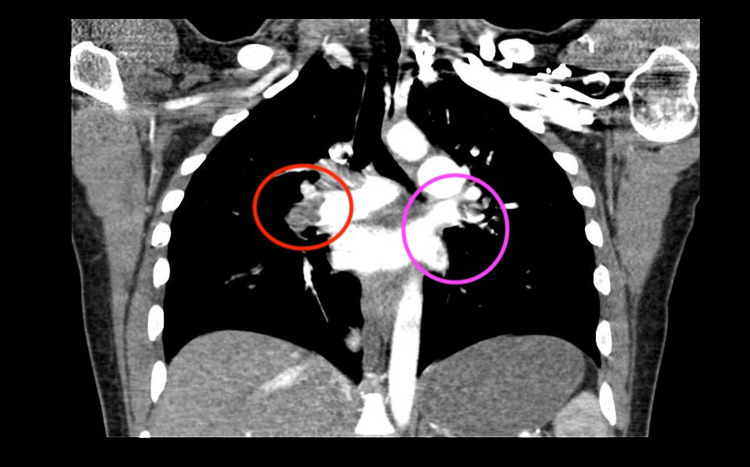
A CT angiogram of the chest (coronal) shows filling defects in the right pulmonary artery (red circle) and filling defects in the left pulmonary artery (pink circle), indicating bilateral pulmonary emboli.

As seen in Figures 1-2, CT-PE showed bilateral acute pulmonary emboli with a large embolic burden.

Her coagulation profile at the time revealed normal prothrombin time (PT) and partial thromboplastin time (PTT) (12 seconds/27 seconds) with an international normalized ratio (INR) of 1.1. The patient was admitted to the medical intensive care unit and started on therapeutic enoxaparin treatment at 1 mg/kg (80mg) every 12 hours. A multidisciplinary approach with the obstetrics and gynecology (OBGYN), surgery, and medicine teams regarding the risk-benefit profile of anticoagulation therapy vs. mechanical thrombectomy was discussed with the patient. However, it was ultimately decided that the patient would benefit most from a mechanical thrombectomy.

Informed consent for the procedure, including risks, benefits, and alternatives, was obtained, and a time-out was performed prior to the procedure. The site was prepared and draped using maximal sterile barrier techniques, including cutaneous antisepsis. Local anesthesia was administered. The right femoral vein was sonographically evaluated and determined to be patent. Real-time ultrasound (US) was used to visualize 19-gauge needle entry into the vessel, and a permanent US image was stored. An 8Fr Cook sheath was placed. The pulmonary arterial system was catheterized using a combination of a GPC, vertebral catheter, Bentson, Amplatz, and 26Fr dryseal sheath. After catheterizing the main pulmonary artery, extensive thrombus in the bilateral proximal pulmonary arteries with decreased pulmonary parenchymal perfusion was noted. With the use of the Inari FlowTriever device and aspiration technique, mechanical suction thrombectomy was performed in the left lower lobar pulmonary artery, right interlobar pulmonary artery, and right upper lobar pulmonary artery. The post-intervention angiography revealed a marked decrease in bilateral pulmonary artery thrombus and a marked improvement in pulmonary parenchymal perfusion. The femoral sheath was removed, and hemostasis was achieved with manual compression. A pursestring suture and a sterile dressing were applied.

Post-thrombectomy, the patient reported improved breathing and was able to ambulate without hypoxia. A repeat echocardiogram post-intervention showed normal right ventricular (RV) size with moderately reduced RV function. The patient was discharged with therapeutic enoxaparin (80 mg every 12 hours) with a plan for close follow-up appointments with maternal-fetal medicine and cardiology.

After discharge, she was continued on anticoagulation throughout the remainder of her pregnancy and the peripartum period and periodically re-evaluated by maternal-fetal medicine, cardiology, and medicine teams to determine ongoing risk factors. At 37 weeks and seven days gestation, she was admitted to labor and delivery for a planned cesarian section with enoxaparin held the night before the procedure. The cesarian section was performed without complications, and she was re-initiated on therapeutic enoxaparin (80 mg every 12 hours) 24 hours post-procedure and continued postpartum for eight weeks of therapy.

## Discussion

Pulmonary embolism is an uncommon and fatal complication of pregnancy. In the United States, 15% of all maternal deaths are due to venous thromboembolism [3]. Confidential inquiries into maternal deaths in the United Kingdom revealed substandard care in more than 50% of all maternal deaths from pulmonary embolisms [4]. With high incidence rates and an increased risk of mortality, extensive care must be taken to ensure early diagnosis and treatment of VTE/PE.

The etiology of VTE/PE directly relates to Virchow’s Triad of hypercoagulability, endothelial damage, and stasis. Venous stasis [5], inherited thrombophilia [5], vascular damage [6], and hypercoagulability [7] have all been studied as root causes for VTE/PE in pregnancy. Recent studies are now discussing the role of socio-economic disadvantages [8], obesity, and other pro-inflammatory diseases [9] in the increase in VTE risk factor analysis. In fact, adults who are socioeconomically disadvantaged and hospitalized with PE exhibit elevated one-year mortality rates in comparison to their non-disadvantaged counterparts, along with 33.3% higher readmission rates within 90 days following PE-related hospitalizations [8].

The classification of PE may vary among most cardiology and critical care associations, but it is clear that hemodynamic instability and right ventricular dysfunction (RVD) are critical components of risk stratification. RV failure is the primary cause of death in PE, and as such, its prognostic value has been studied in both echocardiography and CTPE imaging modalities [10-11].

In cases of hemodynamic instability, especially massive pulmonary embolism, studies have shown that systemic thrombolysis has led to a reduced risk of mortality. Systemic thrombolysis in pregnancy has only been indicated for use in hemodynamically unstable patients due to massive pulmonary embolism [12]. With early use of systemic thrombolysis, it has been shown that hemodynamics, right ventricular dysfunction, and pulmonary pressures have improved benefits versus standard anticoagulation therapy alone. In contrast, submissive and low-risk PE have not yet shown a reduction in mortality, and the role of systemic thrombolysis remains controversial due to the increased risk of bleeding [13].

In regards to contraindications to systemic thrombolysis, consideration should be given to urgent or emergency mechanical thrombectomy. In regards to our case, the patient presented with submassive PE with RVD and hypoxemia. With the relative contraindications of systemic thrombolysis therapy, a multi-disciplinary approach must be taken with patient-oriented goals. To achieve minimal fetal complications and increased positive outcomes, such as reduced cardiac pressures and right ventricular strain, endovascular intervention with mechanical thrombectomy was performed to immediately relieve right heart strain and improve cardiac hemodynamics. Complications that may arise from performing PE catheter thrombectomy include the perforation or dissection of cardiovascular structures, pericardial tamponade, pulmonary hemorrhage, and embolization of the distal thrombus [14].

## Conclusions

Overall, pulmonary embolism is a feared and deadly complication of pregnancy. PE remains a difficult, yet crucial, diagnosis to obtain given the physiological changes of pregnancy and the low index of suspicion seen in clinical practice. Among the many complications that remain within the pregnant population, including socioeconomic, compounding comorbidities, and access to healthcare, the difficulty in diagnosing and treating PE complicates and compounds the underlying challenges in pregnancy. More thorough education needs to be provided to this population, especially with increased risk factors, to aid in the realization of new symptom presentations, allowing patients to present earlier and preventing worsening complications. Treatment with anticoagulation has typically been the mainstay of treatment in the setting of PE/DVT, but more advanced techniques are being studied for immediate and hastened resolution of symptoms, such as mechanical thrombectomy. In addition to this, mechanical thrombectomy provides faster cardiac recovery with improved right ventricular function. Although risks may include radiation and maternal mortality in cases of high-risk pulmonary embolism, they outweigh fetal complications. In the setting of hemodynamic stability and an increased risk of fetal demise due to pulmonary embolism, further discussion and investigation are underway with regard to the use and indication of mechanical thrombectomy. Management continues to be a multidisciplinary approach in order to decide on treatment modalities to preserve both the mother and the baby.
